# Global stabilizing control of large-scale biomolecular regulatory networks

**DOI:** 10.1093/bioinformatics/btad045

**Published:** 2023-01-23

**Authors:** Sugyun An, So-Yeong Jang, Sang-Min Park, Chun-Kyung Lee, Hoon-Min Kim, Kwang-Hyun Cho

**Affiliations:** Department of Bio and Brain Engineering, Korea Advanced Institute of Science and Technology (KAIST), Daejeon 34141, Republic of Korea; Flint Research, Flint Technologies Inc, New Castle County, DE 19808, USA; Department of Bio and Brain Engineering, Korea Advanced Institute of Science and Technology (KAIST), Daejeon 34141, Republic of Korea; Department of Bio and Brain Engineering, Korea Advanced Institute of Science and Technology (KAIST), Daejeon 34141, Republic of Korea; College of Pharmacy, Chungnam National University, Daejeon 34134, Republic of Korea; Department of Bio and Brain Engineering, Korea Advanced Institute of Science and Technology (KAIST), Daejeon 34141, Republic of Korea; Department of Bio and Brain Engineering, Korea Advanced Institute of Science and Technology (KAIST), Daejeon 34141, Republic of Korea; Department of Bio and Brain Engineering, Korea Advanced Institute of Science and Technology (KAIST), Daejeon 34141, Republic of Korea

## Abstract

**Motivation:**

Cellular behavior is determined by complex non-linear interactions between numerous intracellular molecules that are often represented by Boolean network models. To achieve a desired cellular behavior with minimal intervention, we need to identify optimal control targets that can drive heterogeneous cellular states to the desired phenotypic cellular state with minimal node intervention. Previous attempts to realize such global stabilization were based solely on either network structure information or simple linear dynamics. Other attempts based on non-linear dynamics are not scalable.

**Results:**

Here, we investigate the underlying relationship between structurally identified control targets and optimal global stabilizing control targets based on non-linear dynamics. We discovered that optimal global stabilizing control targets can be identified by analyzing the dynamics between structurally identified control targets. Utilizing these findings, we developed a scalable global stabilizing control framework using both structural and dynamic information. Our framework narrows down the search space based on strongly connected components and feedback vertex sets then identifies global stabilizing control targets based on the canalization of Boolean network dynamics. We find that the proposed global stabilizing control is superior with respect to the number of control target nodes, scalability, and computational complexity.

**Availability and implementation:**

We provide a GitHub repository that contains the DCGS framework written in Python as well as biological random Boolean network datasets (https://github.com/sugyun/DCGS).

**Supplementary information:**

Supplementary data are available at *Bioinformatics* online.

## 1 Introduction

Cellular behavior is an emergent phenomenon resulting from complex non-linear interactions between numerous intracellular molecules that can be represented by a Boolean model of a molecular regulatory network. In the Boolean model, nodes represent molecules, links represent physical or functional interactions between molecules, and Boolean logic functions represent non-linear regulatory relationships between molecules (Wang *et al.*, 2012). From the perspective of a dynamic system, the stable cell phenotype can be represented as an attractor of a molecular regulatory network (Huang *et al.*, 2005; Shin *et al.*, 2014), and state transition trajectories to attractors can be represented as an attractor landscape that includes all attractors and their basins (the sets of states converging to the corresponding attractors) (Choi *et al.*, 2012). In the attractor landscape, a disease state is represented by the corresponding abnormal attractor(s) whose basin is enlarged, resulting in increased stability of abnormal attractor(s) (Kim *et al.*, 2013). Therefore, in order to treat diseases, we need network-level analysis to identify optimal control targets for global stabilization that can drive heterogeneous cellular states to the desired phenotypic attractor state with minimal node intervention.

Several previous studies aiming to identify control targets of biological systems utilized only network structure information. For example, the driver nodes that are obtained by structural controllability theory (assuming linear time-invariant systems) are the smallest set of nodes that propagate input signals to all nodes along with the network cascade structure (Liu *et al.*, 2011). Thus, input signals received by the driver nodes can eventually affect all the nodes within a network through cascades. The control effects of driver nodes were experimentally validated using a neural network where the driver nodes determined movement behaviors of *Caenorhabditis elegans* (Yan *et al.*, 2017). Another example, the minimum dominating set (MDS) is the minimal node set, at least one of which is a neighbor of every remaining nodes (non-MDS) (Nacher and Akutsu, 2012). Thus, input signals received by the MDS can be propagated to all nodes through adjacent nodes of the MDS within one step. Nodes in the MDS of human and yeast protein interaction networks were revealed to be enriched in cancer-related and virus-targeted genes (Wuchty, 2014). Although these approaches are advantageous when applied to large-scale biological networks due to their low computational complexity, the control nodes determined by these approaches have been demonstrated to deviate substantially from those determined by analyzing non-linear dynamics, which are biologically plausible. For instance, driver nodes have been revealed to be insufficient to fully control non-linear Boolean networks (Gates and Rocha, 2016), and drug targets are more enriched in control nodes determined by considering non-linear dynamics than in driver nodes (Kim *et al.*, 2013). Moreover, linear and non-linear networks produce opposing results for control importance of a node, which is determined as the probability that a node is in a control node set (Jiang and Lai, 2019). In addition, when controlling a network to a single state, the size of required control nodes is smaller than the size of the driver nodes, since the driver nodes provide full control over the entire network (Wu *et al.*, 2014).

Recently, feedback vertex set (FVS) control was proposed to identify control targets in a network with non-linear dynamics (assuming dissipative systems), which can overcome the limitations of control strategies for linear dynamics (Zañudo *et al.*, 2017). FVS is a set of nodes that make the network acyclic if it is removed. When the FVS of the network is controlled, the rest of the network becomes a tree structure without feedback. As a result, the input signals received by the FVS (including the source nodes) can independently change the state of any node in the network. It has been mathematically demonstrated that controlling the FVS can achieve global stabilization through state transitions to any attractor (Mochizuki *et al.*, 2013). The control effects of FVS were experimentally validated in a gene regulatory network where the FVS determines cell fates of ascidian embryos (Kobayashi *et al.*, 2018). Because this method solely utilizes network structure information, it is called a logic-independent framework.

Although FVS is sufficient to control a system to any desired attractor, it is not a minimal node set for global stabilization to a single desired attractor. Therefore, a control framework that utilizes the actual logics in the non-linear dynamics of biological networks is required to identify biologically plausible and more optimal control targets of biological systems.

Other studies have suggested logic-dependent network control strategies to control biological networks using the non-linear dynamics of the network. Studies employing biological Boolean networks have elucidated the dynamic properties of cellular behaviors, simulated drug responses of cells and identified potential drug targets (An *et al.*, 2020; Choi *et al.*, 2017, 2022; Choo *et al.*, 2018; Park *et al.*, 2020, 2021). Based on the attractor landscape, we developed a framework for the identification of control kernels (CKs)—minimal control node sets for global stabilization (Kim *et al.*, 2013). By controlling the CK, the network state converges from a possible initial network state to the desired attractor. Another example, Zanudo and Albert (2015) defined a stable motif (SM), which is a self-sustaining positive feedback loop for global stabilization, and identified control targets based on the SM. In addition, Yang *et al.* (2018) defined the logical domain of influence (LDOI), a stabilizing effect of nodes and their combinations, to identity control interventions for target control that only drives specific target nodes into their desired states. We also further suggested phenotype control kernels (PCKs)—minimal control node sets for target control (Choo *et al.*, 2018). However, the logic-dependent control frameworks for Boolean networks have limitations when applied to large-scale networks due to their high computational complexity. For example, the SM-finding algorithm involves time-consuming steps such as the construction of an expanded network (a network with additional nodes to represent logical relationships) and network reduction (Zanudo and Albert, 2015). The evaluation of LDOI also requires the generation of an expanded network and uses a heuristic method to identify control node sets (Yang *et al.*, 2018). The CK-identifying algorithm utilizes a brute force search method that requires the computation of state transition graphs for every node set (Kim *et al.*, 2013). Therefore, the development of a computationally tractable and optimal control framework for the global stabilization of large-scale non-linear biological networks remains an open question.

Canalization is central to the approach to study control in Boolean networks. A node with a canalization function has at least one input variable that determines the output state of the node independently of other input variables. Biological networks, including eukaryotic genes regulatory network, are composed of the nodes with canalizing functions (Grefenstette *et al.*, 2006; Harris *et al.*, 2002). Such canalizing functions of Boolean networks were revealed to contribute to preventing chaotic behavior and maintaining stability (Kauffman *et al.*, 2004; Reichhardt and Bassler, 2007; Shmulevich and Kauffman, 2004). Thus, the canalization effect plays an important role in controlling biological networks. A dynamics canalization map was recently developed to quantify these effects in a model and to show the canalization logic of the entire network (Marques-Pita and Rocha, 2013). In addition, it was found that the accuracy of the control framework using only the structural information such as the driver node can be improved by using the canalization information (Gates and Rocha, 2016). However, the canalizing effect of FVS, which is another structural information-based control framework that also applies to non-linear dynamic networks, has not been elucidated.

In this study, we propose a scalable global stabilizing control framework for non-linear complex network represented by a Boolean network utilizing both structural and dynamic information in terms of a canalization effect. First, we analyzed the relationship between CKs and FVSs of biological random Boolean networks. In most cases, FVSs are identified faster than CKs and also include CKs. In addition, we observed that CKs in FVSs act as optimal global stabilizing control node sets for multiple attractors compared to CKs not in FVSs. These findings motivated us to subdivide each FVS to identify CKs that are included in each FVS. Thus, we divided the nodes in each FVS into three subsets by analyzing Boolean dynamics: canalizing sets, canalized sets, and monostable sets. Canalizing sets (optimal FVS subsets for global stabilization) were CKs in most cases and monostable sets (FVS subsets with only one state within each attractor) were generally absent. If present, the monostable set was composed of a small number of nodes for biological random Boolean networks. Based on these findings, we developed a framework of divide and conquer for global stabilization (DCGS) using a network decomposition and control approach based on FVS and canalization. We confirm that our framework has advantages over previous control frameworks with respect to the number of control target nodes, scalability, and computational complexity for biological Boolean networks in the Cell Collective (Helikar *et al.*, 2012). In addition, by analyzing a MAPK network constructed by Grieco *et al.* (2013), we confirm that the DCGS framework is suitable for providing biologically feasible and reasonable control node sets.

## 2 Materials and methods

### 2.1 Boolean network and attractor

In our study, we used a synchronized Boolean network. In the network, the state of a node (0 or 1) at time *T* is determined by its Boolean function, which is composed of the states of the nodes directly upstream at time *T*−1. The state of a network is defined as a vector consisting of the states of all nodes in the network. A state transition graph is a graph that has the state of a network as a node and a synchronous update relationship between states as a directed link. States that make up a cycle (including self-loops) in a state transition graph are referred to as attractors. More specifically, a state with a self-loop is defined as a point attractor, and two or more states that make up a cycle are defined as cyclic attractors. The set of states that finally reach an attractor is referred to as the basin of the attractor and the size of the set is referred to as the basin size of the attractor.

### 2.2. Biological random Boolean network

To generate biological random Boolean networks composed of 10 nodes, we created a network structure with out-degrees that follow a power-law distribution (*P*(*k*)=*Ak*^-γ^) and in-degrees that follow a Poisson distribution (λ = 1.75) (Supplementary Fig. S1). In the power-law distribution, *k* is ranged from 1 to 10, *A* is a constant that ensures that the *P*(*k*) values add up to 1, and γ is about 2.5. γ was set considering that γ from a biological network is between 2 and 3 (Leclerc, 2008). In the Poisson distribution, λ was set to 1.75 considering that the average degree of biological network is about between 1.5 and 2 (Albert and Barabási, 2002). Adopting the Boolean functions already used in the Cell Collective’s biological Boolean networks (Helikar *et al.*, 2012), the Boolean function of a particular node was randomly chosen from among the functions of the node with the same in-degree. For further perform intensive simulation analysis, the biological random Boolean networks with two or more point attractor are selected. Additional information about attractor, basin size, strongly connected components (SCCs), FVS and CK of 100 biological random Boolean networks used in the analysis are provided in Supplementary Table S1. We also provide Boolean logics of each random network in our GitHub repository (https://github.com/sugyun/DCGS). Attractors and the corresponding basin ratios were computed using PyBoolnet Python package (Klarner *et al.*, 2017).

### 2.3 Minimal FVS control framework

We used the brute force algorithm (max_search = the number of nodes in the network) in the CANA Python package (Correia *et al.*, 2018) to find all minimal FVSs. The algorithm increases the size of testing sets from 0 to the number of nodes in the network by increments of 1 until it finds a FVS.

### 2.4 Brute force control kernel framework

The brute force control kernel (Brute force CK) framework searches for CKs by increasing the number of fixed nodes from 0 to the number of nodes in the network by increments of 1, until only the target attractor is left in the state transition graph. The state transition graph is analyzed using the PyBoolnet (Klarner *et al.*, 2017) Python package.

### 2.5 Canalizing effect analysis

To identify downstream nodes that are fixed when a specific node is fixed to a certain state (0 or 1), we computed the canalizing effect of the fixed nodes (Kauffman, 1974). In this procedure, we repeatedly fixed nodes with Boolean functions that were determined by previously fixed nodes until there were no additional nodes to fix.

### 2.6 Strongly connected component hierarchical structure analysis

We defined the hierarchy of SCCs by repeatedly finding and removing the lowest SCCs using the attracting component function in the NetworkX Python package (Hagberg *et al.*, 2008) until all nodes in the network were removed.

### 2.7 Canalizing set searching procedure

When searching for canalizing sets of a SCC without considering monostable sets, we examined the canalizing effect by increasing the size of FVS subsets from 0 to the size of the FVS by increments of 1 until all nodes of the FVS were directly fixed by the canalizing effect.

### 2.8 Divide and conquer framework for global stabilization

The framework of DCGS is composed of two main parts: one involves the identification of the SCC hierarchical structure and all FVSs in each SCC, and the other involves searching for canalizing sets of all FVSs in each SCC without considering monostable sets. When sequentially analyzing a SCC to find canalizing sets, we fixed the upper SCCs of the SCC currently being analyzed. When integrating the analyzed results of each SCC, we only aggregated the smallest canalizing sets.

### 2.9 Stable motif framework

To find control node sets using the SM framework, we used the SM Python code (max_simulate_size = 20, target_method = ‘history’, driver_method = ‘internal’) from the GitHub repository (https://github.com/jcrozum/pystablemotifs) (Rozum *et al.*, 2022).

### 2.10 Biological Boolean network preprocessing and analysis

Among the biological Boolean networks in the Cell Collective (Helikar *et al.*, 2012), we selected 39 networks that have more than two attractors (including at least one point attractor) when all input nodes are fixed with the Off state. We also excluded networks in which we could not search FVSs of each SCC as none of the frameworks worked with such networks. The major point attractors of the 39 networks were used to compare the performances of the network control frameworks. Attractors and the corresponding basin ratios were computed using PyBoolnet Python package (Klarner *et al.*, 2017).

### 2.11 MAPK network model analysis

We used the Boolean logic of the MAPK network downloaded from the Supporting Information (journal.pcbi.1003286.s006) and visualized the MAPK network using the GINsim model file downloaded from the Supporting Information (journal.pcbi.1003286.s004) (Grieco *et al.*, 2013).

## 3 Results

### 3.1 Association between FVS and CK in biological random Boolean networks

**Fig. 1.**
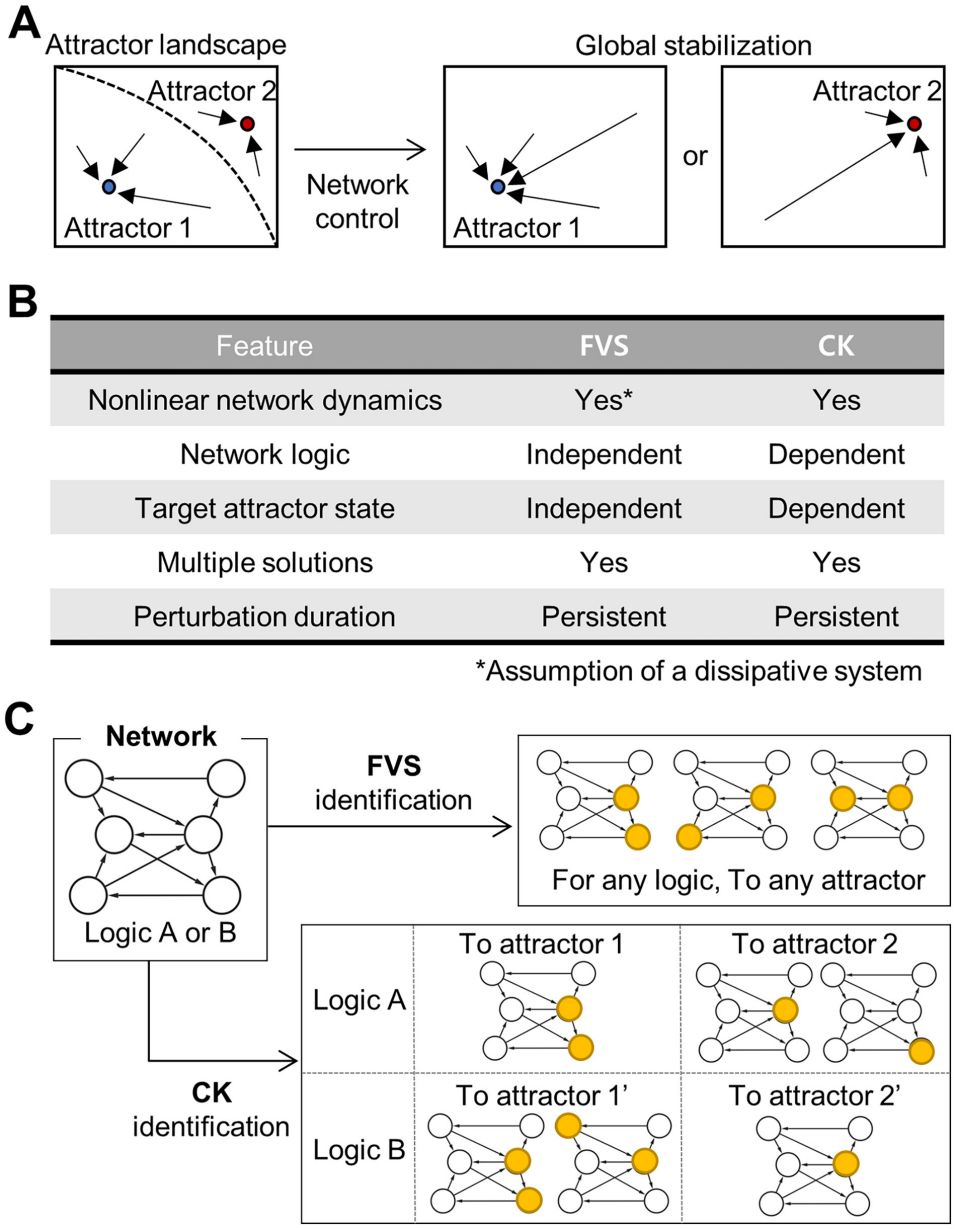
Comparison of network control through FVSs and CKs. (**A**) Attractor landscape changes in global stabilization to one of the two attractors through network control. (**B**) Common and different characteristics of two network control frameworks, FVS and CK. (**C**) Illustration of identified FVSs and CKs for different attractors in the network with different logics. Control nodes identified in each case are shown in yellow

Network control to the desired attractor can be achieved through global stabilization of the attractor landscape (Fig. 1A). In this study, we analyzed two frameworks of network control: minimal FVS, a minimal set of nodes that can remove all cycles of a network (hereinafter, minimal FVS will be referred to as FVS), and CK, a minimal set of nodes for global stabilization (Fig. 1B). They share a common trait in that both can stabilize all states of a network with non-linear dynamics to a point attractor though persistent inputs to control nodes. Also, there can be multiple sets of control nodes with a minimum number to achieve the same control goal. However, unlike FVS, CK depends on the logic of the network and the target attractor (Fig. 1C).

To deeply explore the relationship between FVS and CK in the biological context, we analyzed 263 point attractors of 100 biological random Boolean networks with 10 nodes (Supplementary Table S1). The biological random Boolean networks had scale-free out-degree distribution and a Poisson in-degree distribution (Supplementary Fig. S1; see Section 2 for details) and had Boolean functions that were previously used in the biological Boolean networks of the Cell Collective (Helikar *et al.*, 2012). We obtained FVSs by searching for minimal sets of nodes that render the network acyclic and obtained CKs by searching for minimal sets of nodes that stabilize the network to a target point attractor.

**Fig. 2.**
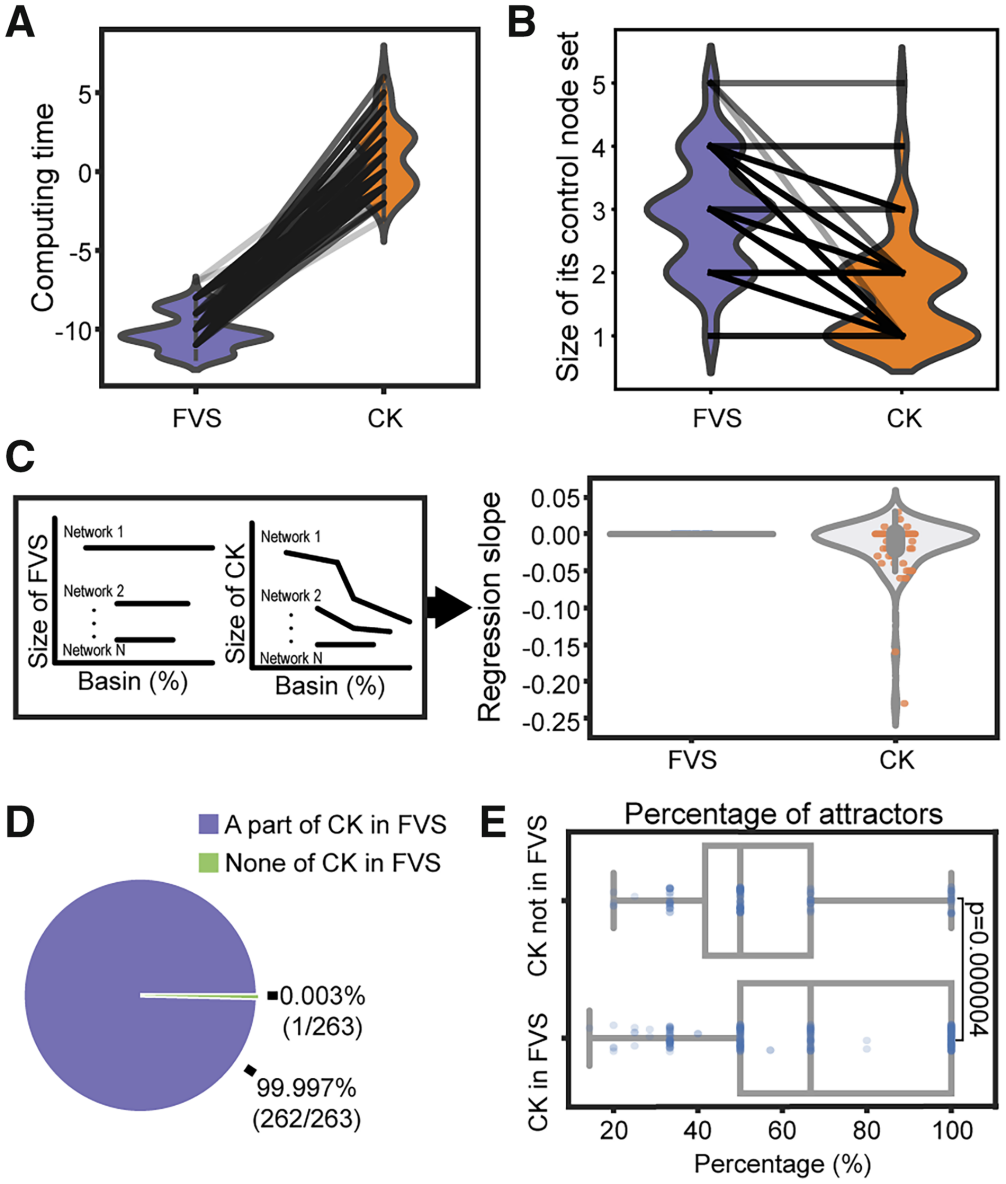
Relationship between FVSs and CKs in biological random Boolean networks. (**A, B**) Comparisons of the computing time (A) and size of sets (B) of FVSs and CKs for 263 point attractors of 100 biological random Boolean networks. Each line connects the results of FVSs and CKs for the same attractor within the same network. The computing time was calculated as log2(time [s] + 0.0001). (**C**) Changes in control node set size according to the basin ratios of target attractors of each network. On the left panel, conceptual line graphs show the relationship between the basin ratio and different sizes of control node set of each network. On the right panel, violin plots show the distributions of first-order regression slopes for FVS and CK. (**D**) The pie chart shows whether at least one of CKs of each target attractor is included within one of FVSs from the network or not. Two hundred sixty-three point attractors of 100 biological random Boolean networks were analyzed. (**E**) Box plots showing the percentage of attractors where CK acts as an optimal global stabilizing control node set. The number of CKs included in FVS is 221, and the number of CKs not included in FVS is 115. A two-sample *t*-test was conducted between the two groups (*P* = 0.000004)

We compared various aspects of FVSs and CKs such as search time, the size of the sets and inclusiveness between FVSs and CKs. In the random biological Boolean networks, FVSs could be rapidly identified as only structural information is required to find FVSs (Fig. 2A). In contrast, CKs can be found with dynamic information obtained via a total inspection of state transition graphs; thus, CKs required a longer search time compared to FVSs (Fig. 2A). Note that all lines that connect the results of FVSs and CKs for the same attractor within the same network in Figure 2A have positive slopes. When we compared the sizes of the sets, FVSs, which are sufficient control targets to drive a network to any attractor, have larger or equal set sizes compared to CKs, which are minimal control targets for each attractor (Fig. 2B). Note that 80% (210 out of 263 attractors) of the lines in Figure 2B have negative slopes. These results suggest that even though FVS can be found quickly, most FVSs are not optimal control node sets for global stabilization to a desired attractor within biological random Boolean networks. In addition, FVSs are constant in size in each network regardless of the target point attractor. Conversely, CKs varied in size according to the target point attractor and exhibited a tendency to decrease as the basin ratio of the attractor increased. Thus, we found that the regression slopes between the basin ratio and the size of CKs tended to be negative (Fig. 2C). We also found 262 out of 263 cases that at least a single CK of a target attractor was a subset of a FVS of the network. Furthermore, the probability that no CK of a target attractor was included in any FVS of the network was only one case out of 263 point attractors (Fig. 2D). Interestingly, CKs that were included in FVSs were found on average to be CKs for 68% of attractors in each network, whereas CKs that were not included in FVSs were found on average to be CKs for 57% of attractors in each network (Fig. 2E). The two-sample *t*-test produced a significant *P*-value of 0.000004. We also conducted simulation analysis on point attractors of biological random Boolean networks of 15 nodes and confirmed the reproducibility of the simulation results from a different size of network (Supplementary Fig. S2A–E).

These findings indicate that FVSs, which can be rapidly found using only structural information, contain valuable CKs that are CKs for multiple attractors in most cases. This motivated us to subdivide the nodes in each FVS and identify CKs included in each FVS.

### 3.2 Classification of FVS according to network dynamics of nodes

**Fig. 3.**
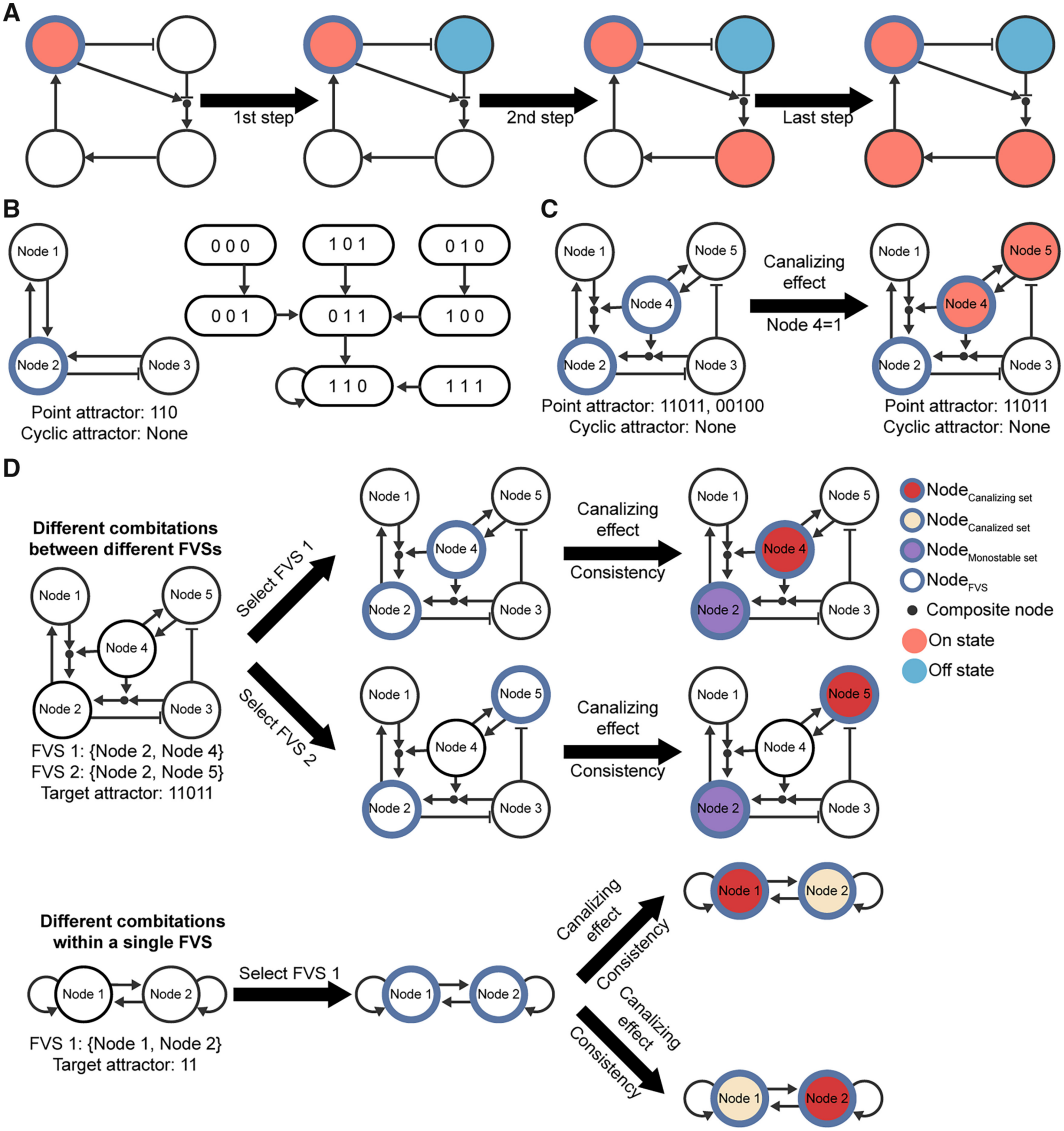
Dividing FVS into combinations of three subsets based on canalizing effect and consistency. The AND logic operator is represented by a composite node (little black dot). The arrow and T bar represent activate and inhibit regulation, respectively. (**A**) An illustration of the process for computing the canalizing effect of a node with the On state. (**B**) Example of a node in a monostable set. A Boolean network is shown on the left and a state transition graph is shown on the right. The Boolean network has only one point attractor (110). Node 2 exhibits consistency and is categorized into a monostable set. (**C**) Example of a node in a conditional monostable set. When the canalizing effect of Node 4 with the On state is reflected, the Boolean network has only one point attractor (11011). Node 2 exhibits consistency when Node 4 is fixed in the On state and is included in a conditional monostable set. (**D**) Examples showing different combinations of canalizing sets, canalized sets and monostable sets between different FVSs (top) and within a single FVS (bottom). Nodes in each specific node set are denoted by different colors and lines

To investigate how control nodes govern entire networks, we evaluated the network dynamics of the nodes in each FVS based on their canalizing effect and consistency. The canalizing effect of a node that is fixed to a constant state (0 or 1) is iteratively determined by the number of downstream nodes that are subsequently fixed to a constant (Fig. 3A; refer to Section 2 for details). The consistency of a node refers to the property of the node to consistently maintain the same value across all attractors without direct fixation (Fig. 3B and C). According to these two aspects, FVS nodes were subdivided into three sets. We defined canalizing sets as minimal subsets of each FVS that must be fixed to control the entire network to a desired attractor. Canalized sets were defined as subsets of each FVS that are directly fixed to a constant by a canalizing set. Finally, we defined monostable sets as subsets of each FVS that exhibit consistency when a canalizing set is fixed. Some nodes of a monostable set have only one state regardless of whether a canalizing set is fixed (Fig. 3B), whereas others only have one state if a canalizing set is fixed (Fig. 3C). Several combinations of canalizing sets, canalized sets and monostable sets can exist among multiple FVSs (upper part of Fig. 3D) or even within a single FVS (lower part of Fig. 3D).

In summary, we were able to divide the nodes belonging to each FVS into combinations of the three subsets (canalizing sets, canalized sets and monostable sets) by analyzing the canalizing effect (which evaluates direct fixation effects) and consistency (which evaluates indirect fixation effects). The canalizing effect of the nodes in the canalizing set can govern the dynamics of the entire network—including the canalized set—to reach the target attractor when the nodes in the monostable set are consistent with the target attractor.

### 3.3 Canalizing sets enriched with CK

**Fig. 4.**
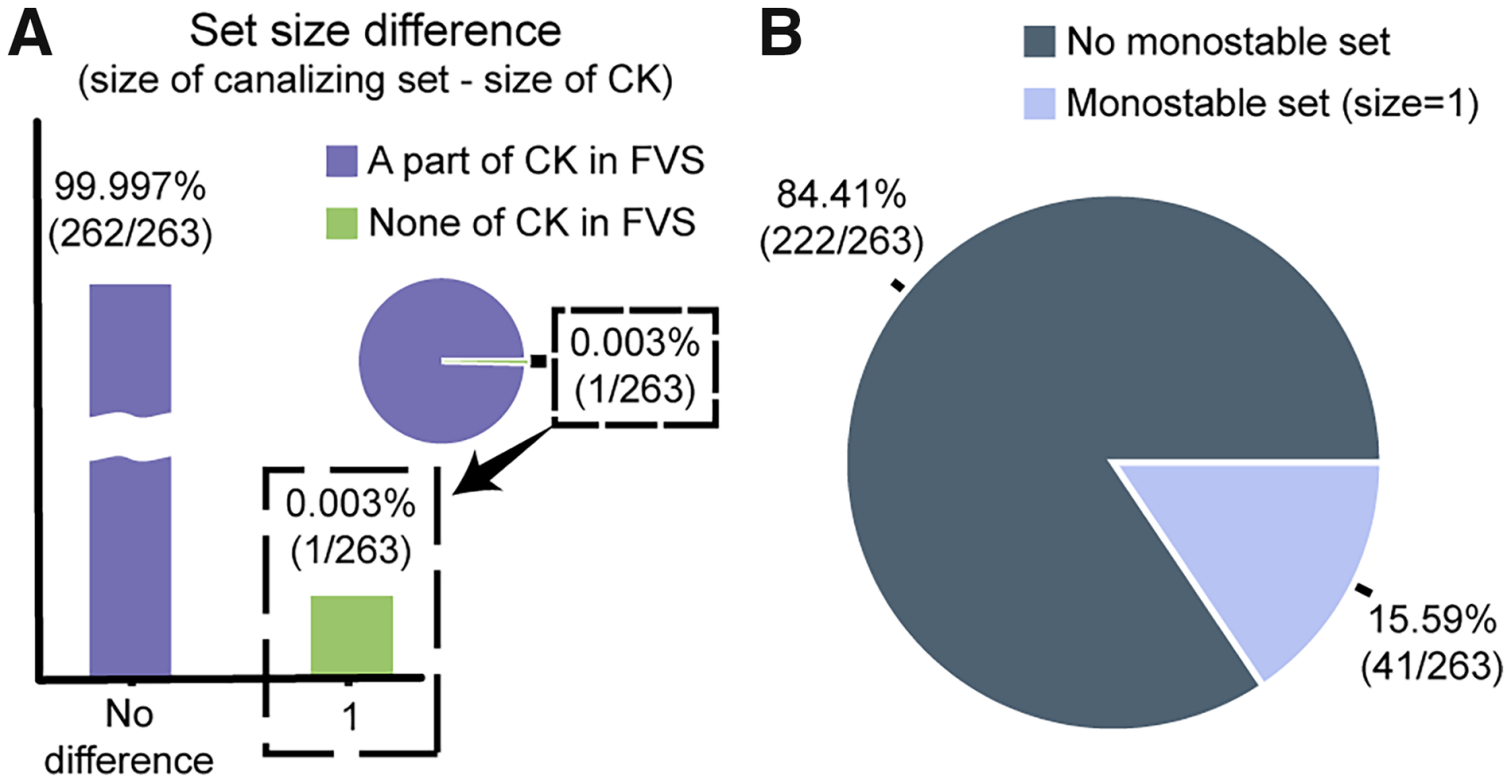
Analysis of FVS subsets in the biological random Boolean networks. (**A**) Size difference between canalizing sets and CKs for 263 point attractors of 100 biological random Boolean networks. (**B**) Pie graph showing the percentages of differently sized monostable sets for 263 point attractors of 100 biological random Boolean networks

In the 263 point attractors of 100 biological random Boolean networks, we divided the nodes in each FVS into combinations of the three aforementioned subsets and analyzed the relationships between the three subsets and CKs (Supplementary Table S2). Canalizing sets were CKs in 99.997% (262 cases) of the attractors, where a portion of CKs of a target attractor are included in any FVS of the network. Thus, canalizing sets were the same size as CKs (the purple bar in Fig. 4A). In 0.003% (one case) of the attractors, where no CK of a target attractor was included in any FVS of the network, canalizing sets were not CKs. However, the canalizing sets were only one node larger than the CKs (the green bars in Fig. 4A). In such rare cases where canalizing sets were not CKs, the CKs from the outside of FVSs directly or indirectly fixed the nodes of the canalizing set (Supplementary Fig. S3). Monostable sets appeared in 15.59% (41 cases) of the attractors (Fig. 4B) and all had sizes of 1. We also confirmed the reproducibility of the simulation results by analyzing biological random Boolean networks with 15 nodes (Supplementary Fig. S2F and G).

In short, canalizing sets of biological random Boolean networks were CKs in most cases, and monostable sets mostly did not exist or appeared in small sizes. Based on these findings, we devised a framework that suggests CKs by finding canalizing sets of each FVS using only the canalizing effect and assuming that there is no monostable set.

### 3.4 Development of a DCGS framework for the global stabilization of large-scale biological Boolean networks

**Fig. 5.**
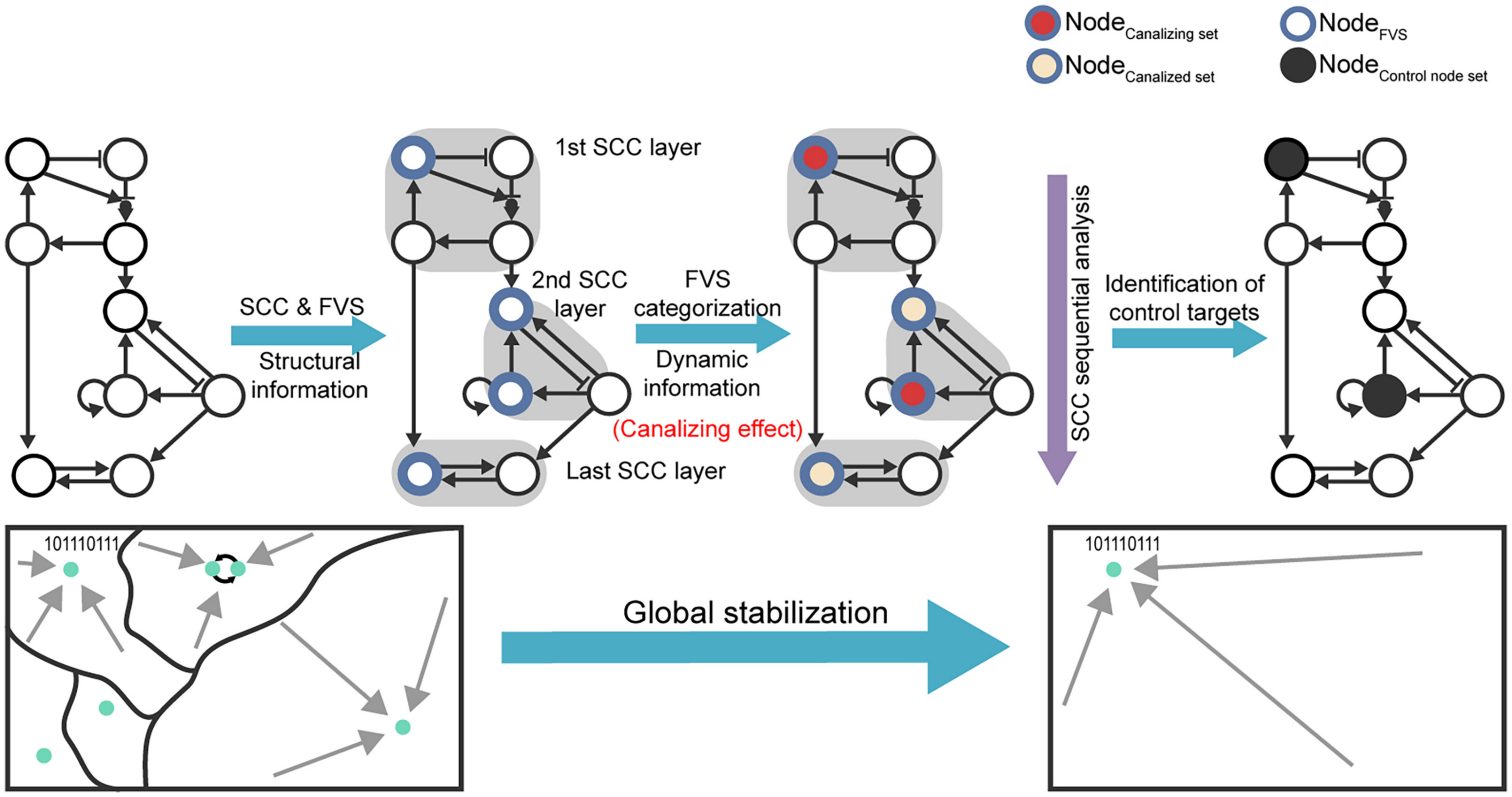
DCGS framework for large-scale biological Boolean networks. The DCGS procedure diagram is shown at the top. First, using structural information, the network is decomposed into SCCs and FVSs are identified for each SCC. Next, using dynamic information, the nodes of each FVS are categorized into canalizing and canalized nodes. SCCs are analyzed sequentially from the top to the bottom. Lastly, control node sets for global stabilization are identified based on the aggregated results of each SCC. Attractor landscapes before and after global stabilization through DCGS framework are shown at the bottom. Nodes in each specific node set are denoted by different colors and lines

Herein, we introduce a DCGS framework that sequentially analyzes and combines small subnetworks to find control node sets for the global stabilization of large-scale biological Boolean networks (Fig. 5).

1. We utilized structural information of a given network and divided the network into hierarchies consisting of SCCs (a subnetwork where every node has a path to every other node). Subsequently, all FVSs were determined for each SCC. By obtaining all FVSs for each SCC and combining them, we achieved results that were identical to those obtained using a brute force approach with the entire network. Moreover, we were able to more rapidly identify all FVSs in large-scale networks.
2. We incorporated Boolean dynamic information. By sequentially investigating from the highest SCC to the lowest SCC, we divided the nodes in each FVS of each SCC into canalizing sets and canalized sets without considering consistency. When we sequentially investigated SCCs, we fixed the nodes included in the upper SCCs of the SCC currently being investigated to a target attractor state. Since the sum of the canalizing effects of each node differs from the canalizing effect of a set of nodes (Supplementary Fig. S4), the canalizing effect was investigated by increasing the size of the FVS subset until canalizing sets were found.
3. We combined the analysis results of each SCC. Among the canalizing sets found in the FVSs of each SCC, we only aggregated the canalizing sets of the smallest sets. To enable others to reproduce the results in Figure 5, we made our Python code available at a GitHub repository (https://github.com/sugyun/DCGS).

### 3.5 Comparative analysis of the DCGS framework and other control frameworks

To compare the developed DCGS framework with three existing frameworks with respect to the applicable network size, the number of nodes in the control node sets, and the computing time, we conducted global stabilization on major point attractors of biological Boolean networks in the Cell Collective (Helikar *et al.*, 2012). We selected 39 biological Boolean networks with more than two attractors (including at least one point attractor) when all input nodes are fixed with Off states. Detailed information about the networks is provided in Supplementary Table S3. The first control framework for comparison was the FVS control (FC) framework (Zañudo *et al.*, 2017), which suggests control node sets composed of input nodes and FVSs. We identified FVSs via a brute force approach by searching minimal sets of nodes that render a network acyclic if absent. Given that the input nodes were already fixed in the Off state, the size of the control node sets suggested by the FC framework only considers nodes in the FVS, excluding the input nodes. The second control framework for comparison was the brute force CK framework, which searches for minimal sets of nodes that stabilize a network to a target attractor. The final control framework was the SM framework, which stabilizes fixed states within a minimal SCC (Zanudo and Albert, 2015). All tests were performed on a server with an Intel(R) Xeon(R) CPU E5-2697 v3 (2.60 GHz, 28 core processor) with 128GB of memory running Ubuntu 16.04.5 LTS and Python v3.6 (64 bit). When the simulation running time exceeded 48 h, the simulation was forcibly terminated and the results were denoted as NA.

**Fig. 6.**
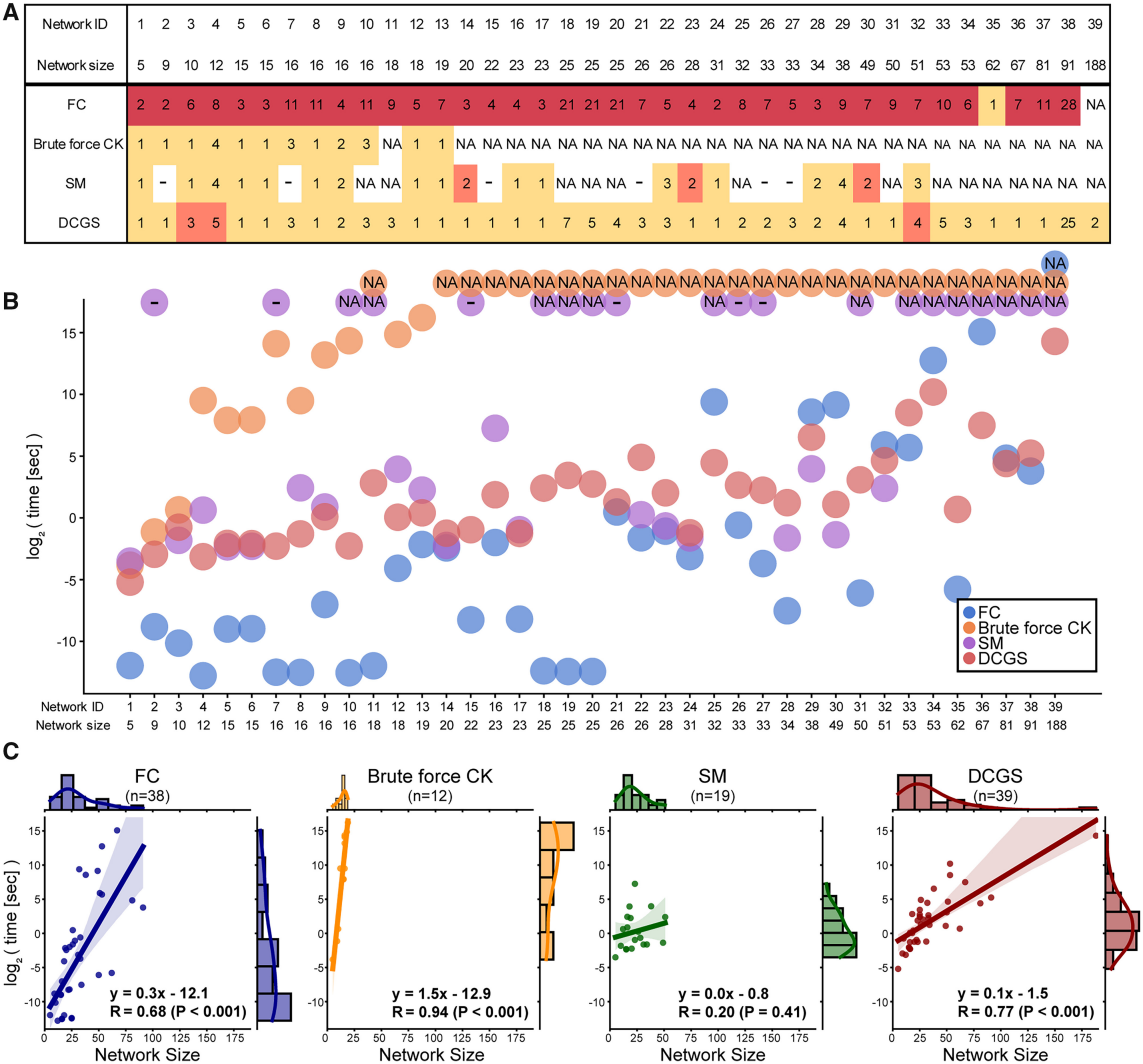
Comparison of Boolean network control frameworks using biological networks in the Cell Collective. (**A**) Sizes of control node sets for global stabilization of major point attractors for 39 biological Boolean networks in the Cell Collective. High, middle and low values in each column are colored red, orange and yellow, respectively. If a framework took more than 48 h, it is denoted as ‘NA’. If a framework suggested incorrect control node sets, it is denoted as ‘-’. (**B**) Computing time comparison. The *x*-axis is the network ID and the *y*-axis is the log2 value of time [s]. Cases denoted by ‘NA’ or ‘-’ are visualized at the top of each network ID. (**C**) Regression plots of four frameworks. The *x*-axis is the network size and the *y*-axis is the log2 value of time [s]. The first-order regression slope, *y*-intercept and spearman correlation coefficient with the associated *P*-value are shown on the bottom right corner of each plot

We investigated the analyzable network size and the size of the control node sets of each control framework (Fig. 6A). The FC framework only utilizes structural information, which allowed the FC framework to obtain control node sets for all network examples except the largest network. However, for the same reason, the FC framework suggested the largest control node set sizes among all frameworks. On the other hand, the brute force CK framework presented minimal control node sets, but the applicable network size was the smallest. Under our simulating conditions, CK could not be computed on networks with more than 20 nodes within 48 h. In addition, due to the large size of the control node set, CK could not be computed even on network ID 11 with 18 nodes within 48 h. The SM framework suggested near-optimal control node sets but failed to be applied to a wide range of networks; there were 15 cases in which the SM framework lasted more than 48 h and six cases in which the wrong control node sets were suggested. Cases in which the control node sets were incorrect are denoted as ‘-’ in Figure 6A. In contrast, our framework was applicable in networks of all sizes and simultaneously provided control node sets very similar to CKs in size. We scrutinized three cases where the DCGS framework suggested control node set sizes that were larger than the sizes suggested by the other frameworks: the networks ID 3 and ID 4 had monostable set sizes of two and one, respectively, whereas the network ID 32 had a conditional monostable set of size one. The FC framework was unable to complete network ID 39 with 188 in 48 h, whereas the DCGS framework was able to do so. This is because the network ID 39 is an unusual network with 103 SCCs, which is suitable for the DCGS framework (Supplementary Table S3).

We also measured the simulation running time of each framework (Fig. 6B and C). Although other frameworks (CK, SM) based on dynamical information were not able to complete simulations on several large networks, the DCGS framework obtained the control targets for all examples. The simulation running time was also relatively short, similar to the FC framework (Fig. 6B). For the FC framework, network size and the log-scaled running time exhibited a high correlation (*R* = 0.68), and the log-scaled running time gradually increased with a slope of 0.3 depending on the network size (Fig. 6C). In addition, due to the nature of the framework to utilize only structural information, the running time was generally short. For the brute force CK framework, network size and the log-scaled running time exhibited the highest degree of correlation (*R* = 0.94). The steepness of the slope between network size and the log-scaled running time indicated that the framework is only applicable for small-scale networks (Fig. 6C). Although the SM framework exhibited a low correlation between network size and the log-scaled running time (*R* = 0.0), the networks amenable to the framework had sporadic sizes, and the *P*-value of the correlations was 0.41 (Fig. 6C). In the case of the DCGS framework, the network size and log-scaled running time exhibited a high correlation (*R* = 0.77). The log-scaled running time slowly increased with a slope of 0.1 depending on the network size, and thus the running time did not increase significantly for networks of larger sizes (Fig. 6C). In the Supplementary Text, we provide detailed explanations about the computational complexity of the frameworks. In summary, the computational complexities of each framework are as follows: *O*(2^*N*^) for the FC framework, *O*(*3^N^*) for the brute force CK framework, *O*((*N + 1*)!) for the SM framework, and *O*(*N*_SCC_*⋅2^N^*^scc^) for the DCGS framework, where *N* is the number of nodes and *N*_SCC_ is the maximum number of nodes among SCCs, which is consistent with the aforementioned simulation running time results.

In conclusion, the DCGS framework, which leverages both structure and dynamics, was applicable to even the largest networks and suggested control node sets that were CKs or very similar to CKs in actual biological network models. Furthermore, the DCGS framework only showed a mild increase in running time as the network size increased.

### 3.6 Biologically feasible control targets in the MAPK network provided by the DCGS framework

**Fig. 7.**
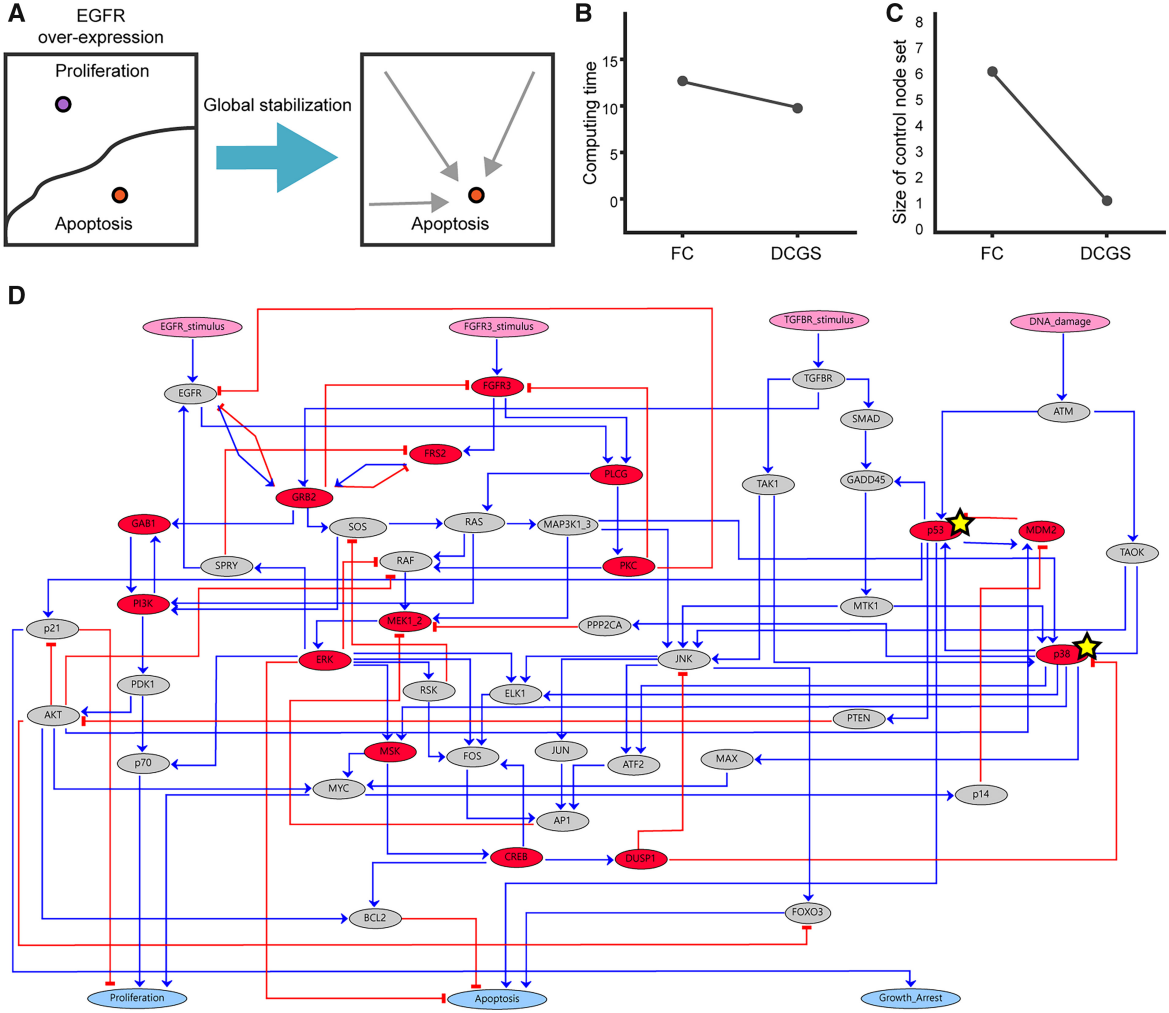
Global stabilizing control of the MAPK network model into the apoptosis attractor. (**A**) Attractor landscapes for the MAPK network following EGFR over-expression before and after global stabilization. When EGFR is over expressed, there are two point attractors: proliferation and apoptosis. (**B**) Computing time comparison between the FC and DCGS frameworks. The *y*-axis is the log2 value of time [s]. (**C**) Size comparison between control node set of the FC and DCGS frameworks. The size of the FVS identified by the FC framework is 6, and the size of the canalizing set identified by the DCGS framework is 1. (**D**) MAPK network where all nodes included in every FVS (FVS nodes) are denoted by red nodes. The number of FVS nodes is 15. The nodes included in every canalizing set (canalizing set nodes) are indicated by yellow stars. The number of canalizing set nodes is 2

To further examine whether the DCGS framework is more suitable for providing biologically feasible and reasonable control node sets than the other frameworks, we applied the frameworks to the MAPK network constructed by Grieco *et al.* (2013). To deal with the state explosion problem with the MAPK network, which consists of 53 nodes, Grieco *et al.* analyzed a reduced MAPK network consisting of 17 nodes. Grieco *et al.* reported that the reduced MAPK network has two point attractors corresponding to proliferation and apoptosis when EGFR is over-expressed and that the reduced network is globally stabilized into the apoptosis attractor when the inhibition of p38 or JNK by DUSP1 is removed. Here, we applied the frameworks to explore control node sets that drive the MAPK network consisting of 53 nodes to the apoptosis attractor when EGFR is over-expressed (Fig. 7A). We subsequently compared the results of the MAPK network to those of the reduced MAPK network.

When we applied the brute force CK and the SM frameworks, the former complete its process within 48 h and the latter suggested incorrectly that there is no node to control. The FC framework completed its determination within a reasonable simulation time (Fig. 7B) and FVSs composed of six nodes were suggested as global stabilizing control node sets (Fig. 7C). The number of nodes included in every FVS (FVS nodes) was 15, and FVS nodes were denoted as red nodes in the MAPK network of Figure 7D. The DCGS framework completed its determination in a shorter amount of time compared to the FC framework (Fig. 7B). This is because the MAPK network consists of 18 SCCs with the largest SCC of 36 nodes, which contributes to reducing the search space of the DCGS. The DCGS suggested two canalizing sets composed of one node (p53 or p38, Fig. 7C). The number of nodes included in every canalizing set (canalizing set nodes) was 2, and canalizing set nodes were marked by yellow stars in the MAPK network of Figure 7D. p38 is a global stabilizing control node in both the reduced and the original MAPK networks. p53, the canalizing set of the MAPK network, is the upstream activator of JNK, which is a global stabilizing control node of the reduced MAPK network. Thus, these results showed that canalizing sets suggested by the DCGS framework are biologically reasonable and feasible. In contrast, the FC framework requires six control nodes, which is more difficult to realize biologically. In addition, the number of FVS nodes (15) is too broad compared to the number of canalizing set nodes, which makes it difficult to experimentally test drug targets. These results reaffirmed that the FC framework and the DCGS framework are scalable and that, in most cases, the size of the control node sets from the DCGS framework are smaller than those from the FC framework (Fig. 6).

As a result, when applied to the MAPK network model, the DCGS framework exhibited better performance than the other frameworks with respect to applicable network size, computing time, control node set size and the biological feasibility of the suggested control node set. To enable others to reproduce the results in Figure 7, we made our Python code available at a GitHub repository (https://github.com/sugyun/DCGS).

## 4 Discussion

Several control frameworks have been suggested to identify control targets in complex networks. Both the FVS and the CK are control frameworks that stabilize all states of a network to a target attractor. However, little is known about the relationship between them and the common underlying mechanisms how FVS and CK govern the entire dynamics of a network. In this study, we found that about 99% of point attractors of biological random Boolean networks had at least one CK that was included in an FVS. In addition, CKs included in one of the FVSs were found to be valuable CKs that were optimal control targets for 60% of the attractors in each biological random Boolean network. According to nodal dynamics, we divided the nodes of the FVSs into combinations of canalizing sets, canalized sets and monostable sets based on the canalizing effect and consistency. From the analysis of biological random Boolean networks, we determined that canalizing sets (optimal FVS subsets for global stabilization) were CKs in most cases and that monostable sets (FVS subsets having the same state across all attractors without any direct fixation) were rarely present or appeared only in small sizes. The absence or small size of monostable sets implies that biological networks may have been evolutionally designed to integrate external stimuli and respond appropriately.

Based on these findings, we devised a DCGS framework called DCGS that is applicable to large-scale biological Boolean networks using structural and dynamic information. The framework sequentially categorized all FVSs of each SCC into combinations of canalizing sets and canalized sets, then aggregated the canalizing sets to provide control node sets that are CKs or similar to CKs in size (Fig. 5). Thus, the DCGS framework can present control node sets that are mostly smaller than the FVSs by subdividing the FVSs. In addition, as the DCGS framework considers only direct fixation effects through the canalizing effect, it bypasses time-consuming steps such as the construction of an expanded network in the SM framework and the examination of state transition graphs in the CK framework. We demonstrated that the DCGS framework is able to identify a minimum set of control nodes in most cases within a reasonable running time compared to existing network control frameworks, including the FC, the brute force CK, and the SM frameworks for biological Boolean networks in the Cell Collective (Fig. 6) (Helikar *et al.*, 2012). Also, the DCGS framework running time showed the mildest increase in simulation running time as the network size increased. These results indicate that the DCGS framework is suitable for identifying control nodes of large-scale biological networks. For biological networks that are composed of several SCCs as like the networks in Figure 6, we presume that even a network of hundreds of nodes can be analyzed within a feasible simulation time. However, it should also be noted that the simulation time of the DCGS can be significantly delayed for some networks of unusual structures composed of one large SCC or having one large FVS that is almost the same size as the SCC, but such cases are very rare in biological networks (see Supplementary Text for details).

The developed DCGS framework has the advantage of being able to analyze entire dynamics without the need for random sampling or reduction of large-scale networks. Many studies that analyzed the dynamics of biological network models have limitations regarding the size of the network model that can be analyzed. Due to these limitations, only a part of the structure of the network could be analyzed by applying reduction methods to simplify the networks, or only a part of the dynamics of a network could be analyzed by applying a heuristic method (Crespo *et al.*, 2013; Grieco *et al.*, 2013; Kim *et al.*, 2013; Soranzo *et al.*, 2012). Therefore, previously identified control targets may be insufficient to control overall network dynamics, or may be just one set of multiple control target sets. Additionally, the targets may vary each time analysis is performed. By analyzing whole network dynamics using the DCGS framework, we can solve these problems and find unidentified optimal control targets in previously constructed networks. We demonstrated that the DCGS framework could identify reasonable and feasible biological control targets by analyzing the large-scale dynamics of the MAPK network, which was incompletely analyzed in its reduced form (Fig. 7) (Grieco *et al.*, 2013). Large-scale network analysis tools like DCGS are expected to be of greater importance in the upcoming era as biological network models will become larger and more sophisticated with the accumulation of vast amounts of data and the invention of new experimental instruments.

Certain control frameworks developed in the past were designed to use infeasible control inputs for nodes. For example, driver nodes require control inputs with values that continuously change over time (Liu *et al.*, 2011), which is difficult to achieve in biological systems. On the other hand, in our framework, the control inputs are persistently fixed until the system reaches the desired attractor, which is more feasible for biological systems. A recent study demonstrated that if control inputs are not persistently fixed for a sufficient duration, then there is a lower probability for the system to reach the desired attractor after control (Choo *et al.*, 2019). Therefore, further study is needed to identify control nodes that incorporate the duration required to reach the desired state.

In this study, we focused on the problem of controlling networks to target point attractors. However, several biological phenotypes are observed as oscillations, which are represented as cyclic attractors (Choi *et al.*, 2012; Klevecz *et al.*, 2008). To address this problem, the previous CK framework identified control nodes for global stabilization to one of the states of a cyclic attractor (Kim *et al.*, 2013). However, this approach transformed the attractor landscape, which might change the selected target state of the cyclic attractor into a point attractor. Although FVSs can drive the system into the original cyclic attractor, this process requires control inputs that continuously vary, which is infeasible for biological applications. Alternatively, control frameworks that use temporary rather than persistent control inputs may cause systems to converge to cyclic attractors while maintaining the original attractor landscape (Baudin *et al.*, 2019; Choo *et al.*, 2019). However, a relatively large number of control nodes or sequential control inputs are required for such control frameworks, which is also infeasible for biological applications. Therefore, a new type of control framework is needed to efficiently control the system into cyclic attractors.

Our framework has a limitation in terms of determining CKs in rare exceptional cases where monostable sets exist or where all CKs are not subsets of FVSs. To deal with these rare cases, it is necessary to investigate the indirect fixation effect of node combinations, which requires the examination of state transition graphs for each node combination (refer to the Supplementary Text). However, this is a time-consuming process. A method capable of quickly exploring indirect fixations due to combinations of nodes that does not resort to state transition graphs would be invaluable in resolving such exceptional cases to our framework.

In this study, a Boolean model was adopted for analyzing and controlling networks. Compared to other continuous dynamic models using ordinary differential equations, which require many parameters to be estimated and thereby result in huge computational complexities for large-scale networks, Boolean models are advantageous for large-scale network simulations since their state values are discretely abstracted while maintaining core dynamics (Choi *et al.*, 2017; Kim *et al.*, 2013). Recently, machine learning has been proposed as a promising method for network analysis (Fan *et al.*, 2020), but it basically generates black box models whose mechanisms are difficult to be interpreted even though explainable AI is being actively investigated. On the other hand, Boolean network models have the advantage of detailed mechanistic understanding based on dynamics analysis as shown in our study.

In summary, by utilizing our findings regarding the relationship between CK and FVS, we proposed a divide and conquer strategy that can determine control node sets for global stabilization in a short time without compromising the dynamics of large-scale Boolean networks. This was achieved by exploring structural information, including SCCs and FVSs, and dynamic information between nodes in each FVS. Our framework narrows down candidates using structural information then identifies control targets using dynamic information. Overall, our method provides insight into how optimal control targets can be identified in large-scale non-linear network models, including synchronous Boolean network models as well as other dynamic models such as asynchronous Boolean network models and ordinary differential equation network models.

## Supplementary Material

btad045_Supplementary_Data

## Data Availability

All data generated or analyzed during this study are included in this published article (and its supplementary files). DCGS framework and biological random Boolean network datasets are provided in a GitHub repository (https://github.com/sugyun/DCGS).
