# Supplementary material for: Global stabilizing control of large-scale biomolecular regulatory networks
Source: Bioinformatics. 2023 Jan 23;39(1):btad045. doi: 10.1093/bioinformatics/btad045 (PMC9891247; doi:10.1093/bioinformatics/btad045)
Supplement: btad045_Supplementary_Data [file btad045_supplementary_data.zip › Bioinfo_Suppl_revision_revision2.pdf]

Supplementary Information

Global stabilizing control of large-scale biomolecular regulatory networks

Sugyun An[1,2,†], So-Yeong Jang[1,†], Sang-Min Park[1,3,†], Chun-Kyung Lee[1], Hoon-Min Kim[1], and Kwang-Hyun Cho[1*]

[†]*Equally contributed first authors*

[1]Department of Bio and Brain Engineering, Korea Advanced Institute of Science and Technology (KAIST), Daejeon 34141, Republic of Korea.

[2]Flint Research, Flint Technologies Inc., New Castle County, DE 19808.

[3]KM Data Division, Korea Institute of Oriental Medicine, Daejeon 34054, Republic of Korea.

[*]**Corresponding Author:** Kwang-Hyun Cho, Ph.D.

Department of Bio and Brain Engineering, Korea Advanced Institute of Science and Technology (KAIST), 291 Daehak-ro, Yuseong-gu, Daejeon, 34141, Republic of Korea; Tel: +82-42-350-4325; Fax: +82-42-350-4310; E-mail: ckh@kaist.ac.kr

This file includes:

Supplementary Figures 1, 2, 3, 4
Supplementary Tables 1, 2, 3
Supplementary Text
Supplementary References

Supplementary Figures

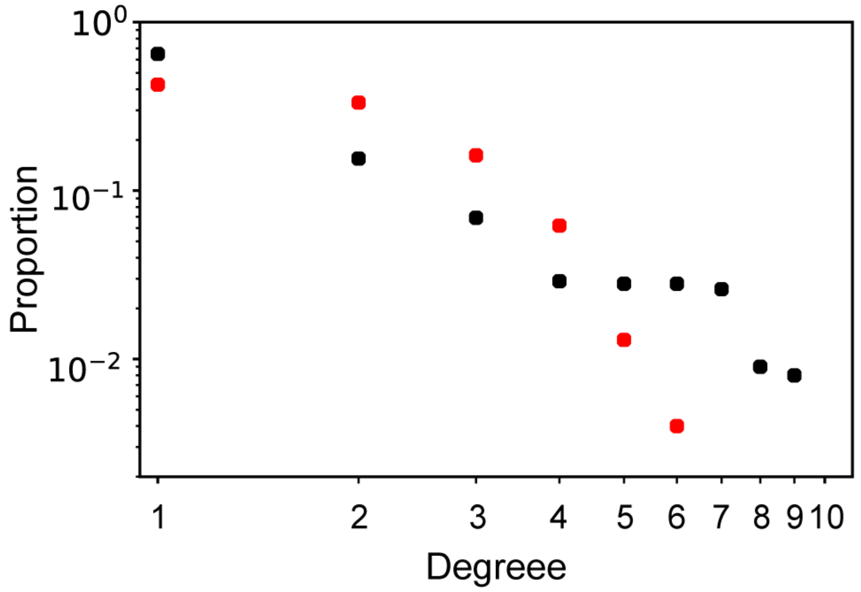

Supplementary Fig. 1 Degree distribution of biological random Boolean networks. In-degree (red circles) and out-degree (black circles) distribution derived from 1000 Boolean update rules of 100 biological random Boolean networks with 10 nodes.

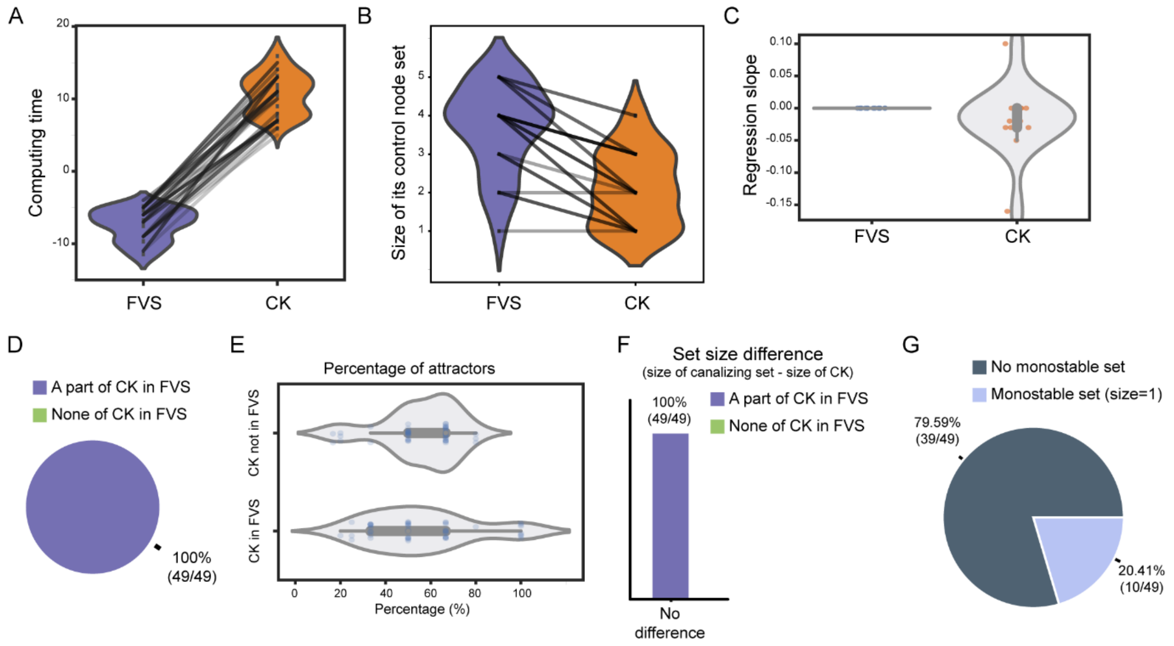

Supplementary Fig. 2. Relationship between FVSs and CKs and analysis of FVS subsets in biological random Boolean networks of 15 nodes. (A-B) Comparisons of computing time (A) and size of sets (B) of FVSs and CKs for 49 point attractors of 15 biological random Boolean networks of 15 nodes. Each line connects the results of FVSs and CKs for the same attractor within the same network. The computing time was calculated as log2(time [sec] + 0.0001). (C) Changes in control node set size according to the basin ratios of target attractors of each network. Violin plots show the distributions of first-order regression slopes for FVS and CK. (D) Pie graph showing whether at least one of CKs of each target attractor is included in one of FVSs of the network or not. (E) Violin plots showing the percentage of attractors where CK acts as an optimal global stabilizing control node set. The number of CKs included in FVS is 48, and the number of CKs not included in FVS is 47. A two-sample t-test was conducted between two groups (p-value = 0.15). (F) Size difference between canalizing sets and CKs for 49 point attractors of 15 biological random Boolean networks of 15 nodes. (G) Pie graph showing the percentages of differently-sized monostable sets for 49 point attractors of 15 biological random Boolean networks.

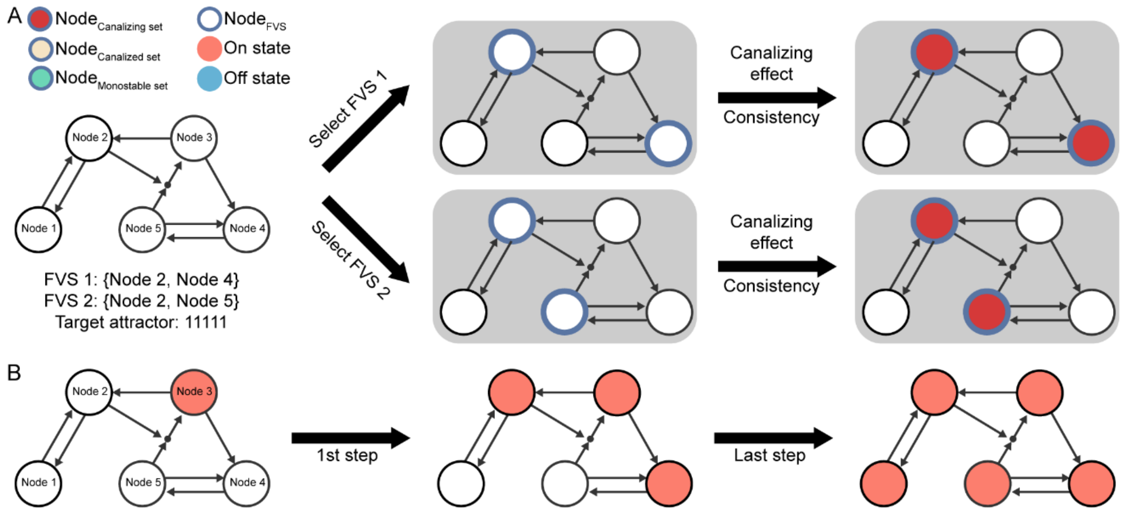

Supplementary Fig. 3. Example case where all CKs exist outside FVSs. (A) Division of nodes in FVSs into combinations of canalizing sets, canalized sets, and monostable sets. Two canalizing sets composed of two nodes are suggested from two different FVSs. (B) The canalizing effect of Node 3 fixed to the On state. All nodes in the network are directly fixed by the canalizing effect of Node 3 fixed to the On state. Node 3 is the CK for the target attractor (11111) and directly fixes the nodes of canalizing sets: both Node 2 and 4, or both Node 2 and 5.

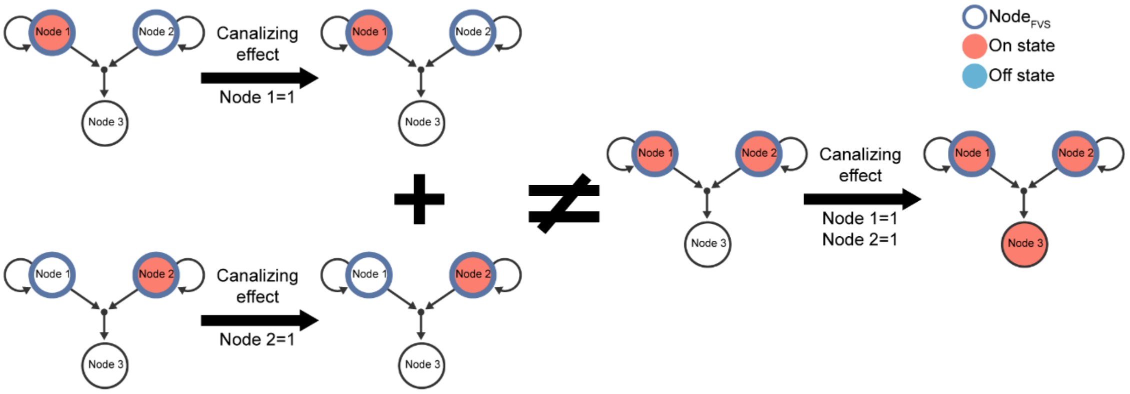

Supplementary Fig. 4. Difference between the sum of canalizing effects of each node and the canalizing effect of a set of nodes. The canalizing effect of each node (Node 1=1 or Node 2=1) is represented on the left. The canalizing effect of the set of nodes (Node 1=1 and Node 2=1) is represented on the right. The sum of the canalizing effects of Nodes 1 and 2 is not equal to the canalizing effect of the node set that includes Nodes 1 and 2.

Supplementary Table 1. FVS and CK simulation results for 263 point attractors of 100 biological random Boolean networks with 10 nodes (Excel file).

Supplementary Table 2. Division of FVSs of 100 biological random Boolean networks into combinations of three subsets (Excel file).

Supplementary Table 3. Detailed information about 39 biological Boolean networks in Cell Collective and simulation results of Boolean network control frameworks for the networks (Excel file).

Supplementary Text

Computational complexity of the FVS control (FC) algorithm

In the FC algorithm, FVSs are found by increasing the size of subsets of nodes in a network by increments of 1. In the worst case where the size of FVS is equal to the number of nodes in a network (N), a total of 2^N subsets must be investigated. Thus, the computational complexity of the algorithm is $O(2^N)$.

Computational complexity of the brute force Control Kernel (CK) algorithm

In the CK algorithm, state transition graphs are investigated by increasing the number of fixed nodes by 1. When investigating ${}_N C_i$ subsets with size i in a network with N nodes, 2^{N-i} initial states are simulated for each subset ($={}_N C_i \cdot 2^{N-i}$). Thus, in the worst case where the size of a control kernel is equal to the number of nodes in a network (N), a total of 3^N ($={}_N C_0 \cdot 2^N + {}_N C_1 \cdot 2^{N-1} + \ldots\ldots + {}_N C_{N-1} \cdot 2^{N-1} + {}_N C_N \cdot 2^0$) initial states are simulated. Thus, the computational complexity of the algorithm is $O(3^N)$.

Computational complexity of the stable motif (SM) algorithm

In the SM algorithm, the most time-consuming process is the generation of an expanded network. The computational complexity of Johnson's cycle algorithm, which generates an expanded network, is known as $O((N + E)(C + 1))$, where N is the number of nodes, E is the number of edges, and C is the total number of directed cycles in G (Zanudo and Albert, 2015). In a fully connected network, E is $\frac{N(N-1)}{2}$ and C is (N - 1)! (Zanudo and Albert, 2015). Thus, considering the worst case, the computational complexity of the algorithm is $O((N+1)!)$.

Computational complexity of the framework of divide and conquer for global stabilization (DCGS)

The DCGS framework is composed of three main parts: an SCC searching part, an FVS searching part, and a canalizing effect investigation part. The computational complexity of the SCC searching part is known to be $O(N+E)$, where N is the number of nodes and E is the number of edges (Nuutila and Soisalon-Soininen, 1994). As described above, the computational complexity of the FVS searching part in each SCC is $O(2^{N_{SCC}})$, where N_{SCC} is the number of nodes in the SCC. In the canalizing effect investigation part, canalizing effects are investigated by increasing the size of FVS subsets by increments of 1. In the worst case, where the size of the FVS and the size of canalizing set are both equal to the

size of the SCC, canalizing effects need to be investigated for $2^{N_{scc}}$ FVS subsets. The maximum number of update steps required to calculate the canalizing effect is N_{SCC}. Thus, the computational complexity to investigate the canalizing effect is $O(N_{SCC} \cdot 2^{N_{scc}})$. Assuming that both the number of SCCs (X) and the number of FVSs (Y) are significantly smaller than N and that N_{SCC} is very close to N, the computational complexity of the DCGS framework is $O(N+E)+O(X \cdot 2^{N_{scc}})+O(X \cdot Y \cdot N_{SCC} \cdot 2^{N_{scc}}) = O(N_{SCC} \cdot 2^{N_{scc}})$.

Complementing exceptional cases of the DCGS framework

If monostable sets are present or if all CKs are not subsets of FVSs, then the DCGS framework suggests larger control node sets than the CKs, albeit without significantly deviating from the CKs. Cases with monostable sets occur more frequently than cases where all CKs are not subsets of FVSs.

Cases where monostable sets exist can be supplemented by sequentially investigating point attractors and cyclic attractors under FVS subset fixation. By first examining the number of point attractors, we can skip the time-consuming cycle attractor identification task if there are two or more point attractors. As most CKs are very similar in size to the canalizing sets, the size of the examined FVS subset must be gradually reduced from the size of canalizing set when examining all possible FVS subsets.

For cases where all CKs are not subsets of FVSs, the canalizing sets were controlled directly or indirectly by CKs outside of FVSs (Supplementary Figure 2). However, it is difficult to find control targets for these exceptions as it requires the examination of direct and indirect fixation effects of all node combinations in each SCC via the inspection of state transition graphs. Such exceptional cases of the DCGS framework could be resolved by developing a method capable of quickly exploring the indirect fixation effects of node combinations without needing to explore state transition graphs.

Supplementary References

Nuutila, E. and Soisalon-Soininen, E.J.I.p.l. On finding the strongly connected components in a directed graph. 1994;49(1):9-14.

Zanudo, J.G. and Albert, R. Cell fate reprogramming by control of intracellular network dynamics. *PLoS Comput Biol* 2015;11(4):e1004193.
